# Hearing Aid Adoption is Associated with the Type of Significant Other in Attendance at Hearing Care Appointments

**DOI:** 10.1177/23312165221131703

**Published:** 2022-11-28

**Authors:** Blair K. Ellis, Gurjit Singh, Stefan Launer

**Affiliations:** 1 Connect Hearing Canada 6221 , Halifax, Canada; 2 Department of Psychology, Ryerson University, Toronto, Canada; 3 Department of Speech-Language Pathology, University of Toronto, Toronto, Canada; 4 Audiology and Health Innovation, Sonova AG, Staefa, Switzerland; 5 School of Health and Rehabilitation Sciences, The University of Queensland, Brisbane, Australia

**Keywords:** hearing care, audiology, hearing aids, significant other, hearing aid adoption, retrospective study

## Abstract

There is increasing evidence to suggest that the implementation of family-centered care practices in clinical audiology yields positive patient outcomes. Previous work showed that significant-other attendance at audiology appointments, a recommended practice consistent with family-centered care, was associated with greater odds of hearing aid adoption and increased satisfaction with hearing aids. The primary goal of this retrospective explorative study was to investigate the unexplored question of whether an association exists between the type of significant other (SO) in attendance at appointments and hearing aid adoption. The study sample consisted of adult patients from a chain of private clinics in the United Kingdom who either attended their audiology appointment with a SO (n = 10,015) or alone (n = 37,152). Six SO types were identified and classified: partner (n = 6,608), parent (n = 76), child (n = 2,577), sibling (n = 208), friend (n = 518), and carer (n = 28). In addition to replicating previous findings which showed that significant-other attendance at audiology appointments was positively associated with hearing aid adoption, results from the current paper also revealed that the odds of hearing aid adoption were greater if the SO was of a stronger relationship tie (i.e., partners, parents, children, and siblings) and not a weaker relationship tie (i.e., friends, carers). These findings suggest that an extension of the non-audiological factor of significant-other attendance during the hearing rehabilitation process should be considered: the relationship type patients have with their significant others.

## Introduction

Hearing loss is a significant global health issue that affects an estimated 1.5 billion people to some degree (WHO, 2021), and the prevalence of hearing loss increases with age (Lin et al., 2011; WHO, 2021). Given that the global population is aging, hearing health care professionals are bracing for a significant increase in demand for rehabilitation services for age-related hearing loss in the coming years. The selection and prescription of hearing aids is often a key component of audiological rehabilitation in hearing healthcare, and hearing aids are commonly recommended as a first-line treatment to prevent the negative consequences of untreated hearing loss. A person's ability to detect, identify, and localize sounds, as well as to recognize speech sounds, are reduced by uncorrected hearing loss (Arlinger, 2003). These auditory deficits are related to various challenges for them such as poorer quality of life (Chia et al., 2007), social isolation (Ramage-Morin, 2016), depression (Mener et al., 2013), functional and cognitive impairments (Williams et al., 2020), and to burden for significant others (Scarinci et al., 2012). That said, the rate of hearing aid adoption is reportedly low (e.g., Abrams & Kihm, 2015; Bainbridge & Ramachandran, 2014), and there is a need to develop a better understanding of what drives patient behavior when making choices about pursuing rehabilitation with hearing aids or not. Notably, there is no universally accepted definition of hearing aid adoption, which can potentially refer to the initial uptake or purchase of hearing aids, or it could refer to the continued use of hearing aids after initial uptake or purchase of the devices. Both are critically important factors to better understand. For this paper, hearing aid adoption refers to the initial uptake or purchase of hearing aids.

While a review of the barriers for, and predictors of, hearing aid adoption is beyond the scope of the present paper, the focus here is on an emerging research theme with a family-centered care perspective that focuses on the influence of significant other (SO) attendance at audiological appointments. Evidence suggests that not only is hearing aid adoption significantly more likely for patients who attend their audiology appointment with a SO (Singh & Launer, 2016), but they also report greater hearing aid satisfaction when support from their SO is evident (Singh et al., 2015). Not surprisingly then, evidence also suggests that positive support from SOs is significantly associated with successful hearing aid use (Hickson et al., 2014). Despite these promising findings, little is known about the identities of the SOs attending audiological appointments and how the attendance of different types of SOs might influence hearing aid adoption. The current paper directly addresses the question of whether the type of SO relationship influences hearing aid adoption by retroactively examining a large sample of patient data from a private chain of audiology clinics in the United Kingdom, where patients either attended an appointment with their SO or alone, and where type of relationship is reported.

### Hearing Aid Adoption and Significant Other Involvement at Audiology Appointments

The involvement of family members in healthcare appointments is a well-known component of patient- and family-centered care (Gerteis et al., 1993). With family-centered care, the clinician, patient, and their family member(s) work together using open communication and shared-decision making strategies to positively influence clinical outcomes (Scarinci et al., 2013).

Jorgensen and Novak (2020) analyzed MarkeTrak 10 data and showed that 78% of people reported that family members informed them that they may have hearing loss, and evidence suggests that SOs are a common prompt for patients to consult a hearing healthcare professional (e.g., Mahoney et al., 1996). This is not surprising since the overall functioning of a SO in their daily life is, in part, influenced by experiences stemming from the patient's hearing loss. In other words, SOs can develop a third-party disability because of a patient's hearing loss (Kamil et al., 2015; Scarinci et al., 2012). In the context of audiology appointments specifically - which primarily focus on helping patients to overcome auditory communication barriers - SO involvement can directly impact clinical processes and outcomes, because SOs are also affected by the communication barriers experienced by the patient (Ekberg et al., 2015).

Not only are patients more likely to adopt hearing aids if they attend appointments with a SO (Singh & Launer, 2016), they also report greater hearing aid satisfaction when they also report greater availability of social support (Singh et al., 2015). Such evidence is notable because many patients do not intrinsically demonstrate autonomous motivation (Ridgway et al., 2015), or the combination of a self-perception of hearing loss and motivation for behavior change (Jorgensen & Novak, 2020; Palmer et al., 2009) – factors which are known predictors of hearing aid adoption. For these patients, it is plausible that family-centered approaches to audiological care that include SO involvement may improve patient self-perceptions regarding the impacts of hearing loss, and increase motivation for behavior changes that could lead to increased likelihood of hearing aid adoption.

The trans-theoretical model (TTM) of behavior change (Prochaska & Velicer, 1997) has been used to describe the findings from a systematic review on audiological and non-audiological determinants of hearing aid adoption (Ng & Loke, 2015). A patient's hearing healthcare journey can be described along the Prochaska and Velicer (1997) six stages of the TTM, from pre-contemplation (i.e., no intent for behavior change), contemplation (i.e., considering a behavior change), preparation (i.e., preparing to make a behavior change and seeking information), action (i.e., making behavior change), maintenance (i.e., maintaining changed behavior), and finally termination (i.e., achievement of self-efficacy with changed behavior). Results from the review suggest that support from SOs during the preparation stage of the TTM may be of importance for hearing aid adoption and use (Table 1, adapted from Figure 2 in Ng & Loke, 2015). The preparation stage likely occurs during a patient's daily life, but it also occurs in the clinical context of a first-time audiology appointment. These appointments are when hearing loss may be identified for the first time, and HA(s) may be recommended.

**Table 1.** Determinants of Hearing aid Adoption Across the First Five Stages of the Trans-Theoretical Model of Behavior Change (Adapted from Figure 2 in Ng & Loke, 2015).

	Audiological	Non-audiological
1. Pre-contemplation	Severity of hearing loss	
2. Contemplation	-	Self-perceived hearing problem
		Expectation
		Demographics
3. Preparation	Type of hearing aid	Group consultation
		Support from significant others
4. Action	Background noise acceptance	-
	Insertion gain	
5. Maintenance	-	Self-perceived benefit
		Satisfaction

### Relationship Status and Social Influence

As research on SO involvement at audiology appointments moves forward, important insights may be drawn by examining questions commonly asked by clinicians, such as: 1) “who are the SOs that attend audiology appointments?”, 2) “does SO type matter for patient outcomes?”.

The importance of such questions has recently been addressed in part by qualitative research examining the role of adult children during their parents’ auditory rehabilitation process (Heacock et al., 2019). It was reported that adult children generally viewed their parents as positive influencers of hearing aid adoption toward each other, but as adult children themselves, they didn't feel that they provided influence toward their parents’ hearing aid adoption (Heacock et al., 2019). This evidence suggests that when elucidating the mechanisms of hearing aid adoption, it may also be relevant to capture variability regarding the type of SO in attendance at audiology appointments, which may be a potential marker of the strength of social influence.

In social network research, social relationships can be defined in terms of the strength of a tie (i.e., “significance or intensity of relationships” (Aral & Walker, 2014, p. 1353)), where weak ties tend to facilitate the connection with more distant social clusters, and strong ties provide more direct social and emotional support (Granovetter, 1983; Marsden & Campbell, 2012). It could be argued that the significant association between hearing aid adoption and the presence of SOs at audiology appointments reported in the literature might be explained by the social influence that the presence of individuals with strong relationship ties have on patient decision making in that particular clinical context. A recent large-scale networked experiment using data from Facebook.com estimated two quantities of product adoption behavior – influence-based adoption, and spontaneous adoption – the former being of particular interest to the current topic of hearing aid adoption (Aral & Walker, 2014). Results showed that greater social influence for product adoption was associated with tie strength measures such as having institutional affiliations in common (125% more social influence by an individual for every institution shared in common with a peer, and a 1355% increase in influence when attending the same college compared to different colleges). Additionally, individuals living in the same current town as their friends showed 622% more influence on those friends, which may suggest that recency of shared social contexts could be relevant for influence. It was also shown that social influence was not moderated by tie strength measures that were associated with common interests. Aral and Walker (2014) report that greater social influence for product adoption was also associated with greater structural embeddedness (i.e., “extent to which individuals share common peers” (Aral & Walker, 2014, p.1353)). Altogether, the results from work on influence and tie strength raise the possibility that audiological outcomes may depend in part on the nature of the relationship between audiology patients and their appointment-attending SOs.

### The Current Research

The primary goal of the current research is to determine whether there is an association between the type of SO in attendance at patients’ first-time audiology appointments, and hearing aid adoption. It is hypothesized that SO type will be significantly associated with hearing aid adoption. Furthermore, given that strong relationship ties have been associated with greater social and emotional support (Granovetter, 1983), and that stronger, compared to weaker ties, exert more influence on product adoption behaviors within social networks (Aral & Walker, 2014), it is expected that greater hearing aid adoption will be observed when patients attend appointments with SOs that are assumed to be a stronger relationship tie (e.g., partner, child, parent, sibling) than of a weaker relationship tie (e.g., carer, friend). The secondary goal of the current research is to replicate findings from Singh and Launer (2016) which found that: 1) the likelihood of hearing aid adoption is greater when patients attend their audiology appointment with an SO compared to attending alone; and 2) a significant interaction between SO attendance by severity of hearing loss exists, whereby SO positively influences hearing aid adoption for patients with mild and moderate hearing losses, but does not have an effect for more severe hearing losses. This explorative study was conducted by analyzing existing, non-overlapping, data from the same network of audiology clinics used for Singh and Launer (2016), where patients were encouraged to bring SOs along to audiology appointments.

## Methods

### Procedures

#### The Nature of the Data

This study consists of a retrospective examination of 47,167 patient records obtained from a private chain of audiology clinics in the United Kingdom that were offering and advertising free hearing tests between February 2014 and November 2016. In the United Kingdom, citizens with aidable hearing losses are eligible to receive hearing aids for free through the National Health Service (NHS) which is a publicly funded healthcare system. Some individuals opt to pursue hearing aid purchases via private clinics for different reasons, including being able to select hearing aid models and styles not available via the NHS, and faster accessibility to services. The measurement variables included information collected at each patients’ first-time appointment at an audiology clinic. The data contained factors such as: demographics (e.g., age, sex, etc.), clinical audiological profile (e.g., audiometric hearing loss), rehabilitation outcome decision (e.g., if hearing instruments were adopted, number of devices obtained), and other factors of interest to this paper, namely whether a patient attended the appointment alone, or with a SO. For those patients who attended with a SO, clinicians had the option to indicate using a free text data entry field, the type of relationship the SO reported with the patient (e.g., child, partner, friend, etc.). Free text data is often very messy to manage due both to typos and the numerous ways clinicians might decide to indicate the SO relationship type (e.g., “friend” versus “attended with a friend from church”). For the current paper, clinician indications of SO relationship type were classified for each patient using custom code written in R (R Core Team, 2020). Data preparation, inclusion criteria, and the classification procedure are described in detail below.

#### Initial Data Preparation

Prior to preparation, the data contained 192,523 patient records. Each time a patient attended an audiology clinic, a patient record was created containing multiple measurement variables, described above. Data were imported into R for processing (R Core Team, 2019). A nine-step procedure based on inclusion criteria was followed to arrive at the final analytical sample, beginning by retaining only the first appointment for each patient who attended more than one appointment (n = 188,518). Next, because data were extracted from the same database used for Singh and Launer (2016), only patient records that had not been previously analyzed were retained (n = 129,293). Only those patients who were recommended a hearing aid based on clinical evidence of aidable hearing loss were retained (n = 97,046). Patient records were retained for all individuals between the ages of 18 and 100 years of age (n = 96,843). Next, data were processed to determine if patients reported experience with wearing at least one HA, and only patients with no experience were retained (n = 51,651). Only patients who disclosed their sex as either male or female were retained so as to remain consistent with Singh & Launer (2016) (n = 49,063; 2,575 participants were reported as “undisclosed” and were removed as they would have been considered missing data in logistic regression models; one participant was reported as transgender and was removed as this was a single occupant category, and 12 participants were removed due to missing responses). All data were retained for patients who either had (n = 2,829) or did not have (n = 46,132) available audiometric data. All individuals who had audiometric data (n = 2,829) were classified using the WE4PTA (i.e., the mean pure-tone average for audiometric thresholds at 500 Hz, 1000 Hz, 2000 Hz and 4000 Hz, for the worse ear, as in Popelka et al. (1998)). Patients either had mild (n = 894), moderate (n = 1,558), or severe (n = 377) hearing loss. Some individuals were classified as “normal hearing” despite being recommended a hearing aid (n = 102), and only those with mild, moderate, or severe loss were retained along with those who had no audiometric data available (n = 48,961). Finally, using custom written code in the R Studio environment (R Core Team, 2019), the type of SO relationship was classified based on the free text clinician descriptions entered into the patient database at each appointment. Only data classified during this procedure were retained (n = 47,471). Finally, only those patient records that were classified as either being “alone”, or with an SO who was either a “Carer”, “Child”, “Friend”, “Parent”, “Partner”, or “Sibling” were retained (n = 47,167).

#### Classification of Significant Other Relationship Types

The primary variable of interest for the classification procedure was a free text column of a spreadsheet, where clinicians either identified whether a patient attended the appointment alone, or they identified the relationship between the patient and the person(s) who accompanied the patient to their appointment (e.g., friend, brother, spouse, etc.). The intent of the classification was to classify the presence or absence of a SO at the appointment, as well as the relationship of the SO with the patient. To perform this classification, nine relationship-type “tags” were identified as follows: “And”, “Carer”, “Child”, “Family”, “Friend”, “Parent”, “Partner”, “Semi-attend”, and “Sibling”. The purpose of conducting this classification was to create a final analytical dataset by excluding all participants in the dataset that did not meet a clear relationship-type classification.

These relationship-type “tags” (see Table 2) are self-explanatory except for four, which are defined here. The tag “And” consisted of all rows in the dataset that indicated that the patient attended the appointment with more than one SO (e.g., “brother, sister, and cousin”). This tag was used as a simple way to identify and exclude all patients who brought more than one SO to their appointment. The tag “Semi-attend” consisted of all rows in the dataset that indicated some information about the partial attendance of a SO, and this tag was used to exclude all of these patients. The tag “Family” was used to parse out all rows where the term “family” alone was used to describe a patient's SO who attended their appointment, and other instances of less common family members (e.g., aunt, uncle, cousin, etc.). This tag was used to exclude all rows from the dataset that were family members not already uniquely tagged above. The tag “Carer” was used to classify patients who attended the appointment with a support worker, caregiver, or carer, (i.e., non-familial support worker) and these cases were retained. The classification procedure was conducted using custom code in R (R Core Team, 2019).

**Table 2.** Description of SO Relationship Tags for Patients Retained or Excluded from the Analysis.

Tag	Definition	n	Retained (Y/N)
Alone	No SO	37152	Y
Carer	A non-familial caretaker	28	Y
Child	Their child	2577	Y
Friend	A friend	518	Y
Parent	A parent	76	Y
Partner	A partner	6608	Y
Sibling	A sibling	208	Y
And	More than one SO	147	N
Family	Any non-tagged family member	112	N
Semi-attend	A SO who partially attended	45	N

#### Sample Characteristics

**Figure 1.**
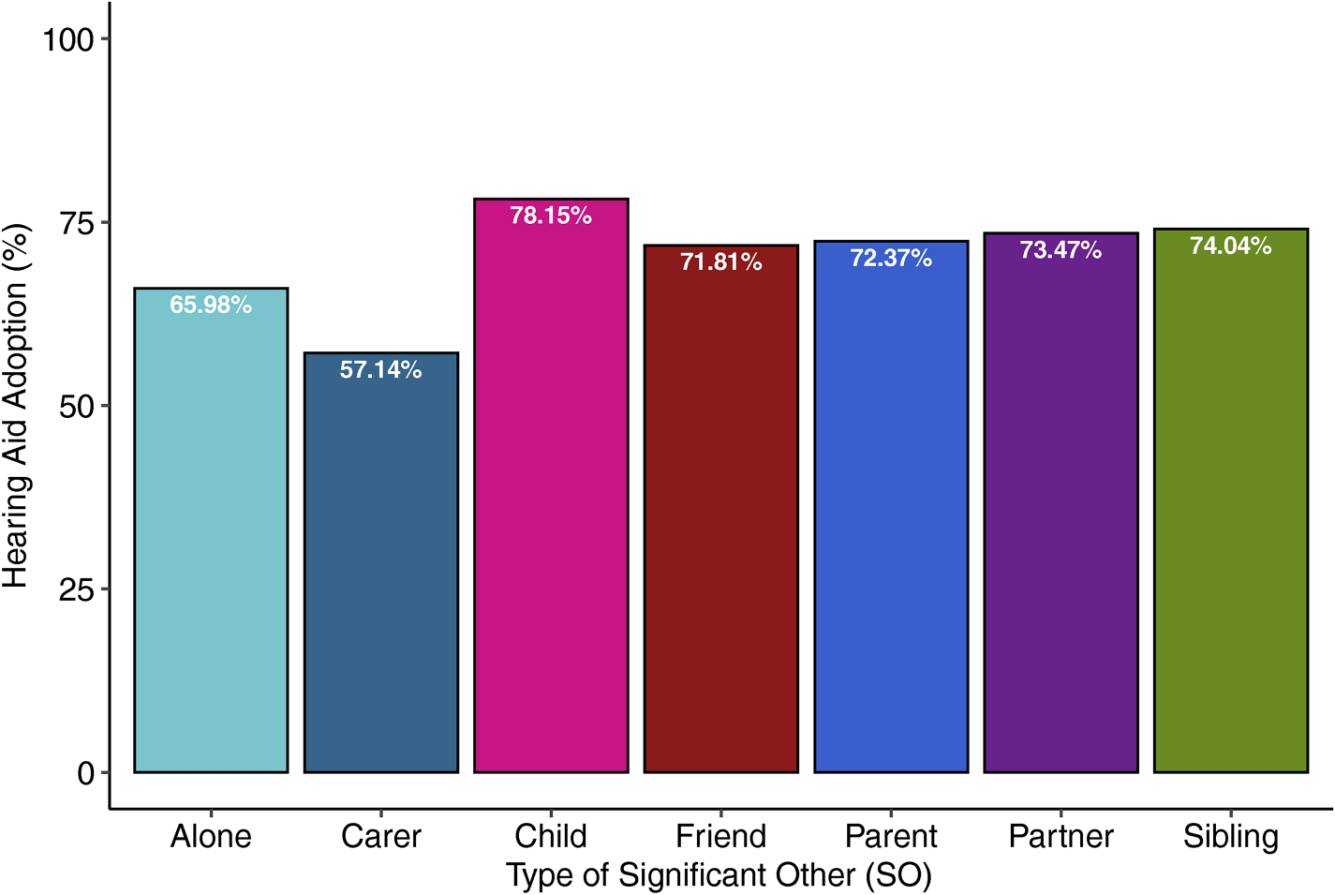
Hearing aid adoption (%) depicted for persons attending appointments by themselves or with different types of significant others.

Demographic information for the final sample of 47,167 participants is provided in Tables 3, 4 and 5. Overall, 21.23% of the sample attended an audiology appointment with a SO (Table 3), which is a substantially lower proportion than the 44.30% reported in Singh & Launer (2016). In this sample, 67.80% of patients chose to adopt a hearing aid after receiving a clinical recommendation, and the proportion of hearing aid adoption for patients attending appointments by themselves or with different types of significant others are show in Figure 1. Independent samples *t* tests revealed that patients in the SO condition were significantly older, more likely to be male (based on proportions), and more likely to adopt hearing aids than those who attended audiology appointments alone (Table 3). Table 4 shows sample characteristics with respect to SO type. Audiometric hearing loss data was only available for a subsample of the data (n = 2,746), and the details of sample sizes across degree of hearing loss, adoption, and SO attendance are provided in Table 5. The degree of hearing loss was calculated using the WE4PTA classification from Popelka et al. (1998). Independent samples *t* tests with Sidak-Bonferroni corrections (Šidák, 1967) were calculated and revealed no significant differences in WE4PTA for any of the subgroups (Table 5).

**Table 3.** Sample Characteristics with Respect to Alone and SO Conditions.

	Alone	SO	t	df	p
Sample size	37,152	10,015	-	-	-
% Female (patients)	52.61%	51.14%	26.58	47,132	p < 0.0001
Mean patient age (years; SD)	70.88 (11.43)	73.12 (10.75)	18.3	16,625	p < 0.0001
% Adoption (patients)	65.98%	74.55%	31.35	29,394	p < 0.0001
Patient adopter mean age (years; SD)	72.06 (11.26)	73.86 (10.64)	12.63	12,964	p < 0.0001
Patient nonadopter mean age (years; SD)	68.59 (11.40)	70.98 (10.78)	10.07	3,787.60	p < 0.0001

*Note.* SD = standard deviation; SO = significant other.

**Table 4.** Sample Characteristics with Respect to SO Type.

	Alone	Carer	Child	Friend	Parent	Partner	Sibling
Sample size	37152	28	2577	518	76	6608	208
% Female	52.61%	60.71%	77.96%	84.17%	69.74%	37.06%	75.96%
Patient mean age (years; SD)	70.88 (11.43)	74.90 (21.87)	80.03 (9.67)	75.36 (10.23)	47.95 (15.83)	70.59 (9.39)	71.68 (12.35)
% Adoption	65.98%	57.14%	78.15%	71.81%	72.37%	73.47%	74.04%
Adopter mean age (years; SD)	72.06 (11.26)	77.83 (21.74)	81.06 (8.95)	76.45 (9.83)	46.23 (16.60)	71.02 (9.24)	72.18 (12.56)
Non-adopter mean age (years; SD)	68.59 (11.40)	71.01 (22.34)	76.32 (11.15)	72.51 (10.71)	52.31 (12.91)	69.38 (9.72)	70.26 (11.74)

*Note.* SD = standard deviation; SO = significant other.

**Table 5.** Mean WE4PTA dB Hearing Loss (HL) for Adopters and non-Adopters for Alone and SO Conditions.

	Degree of HL	Alone	SO	t	df	p
Adopters: Mean WE4PTA (WE4PTA; SD)	Mild	34.73 (4.05) [n = 493]	34.79 (3.96) [n = 118]	−0.14	180.19	0.89
	Moderate	49.52 (5.43) [n = 991]	49.44 (5.48) [n = 271]	0.21	425.88	0.83
	Severe	71.83 (10.99) [n = 255]	70.42 (10.02) [n = 77]	1.05	135.90	0.30
Non-adopters: Mean WE4PTA (WE4PTA; SD)	Mild	34.00 (3.99) [n = 246]	33.98 (2.51) [n = 16]	0.02	20.3	0.98
	Moderate	47.90 (4.86) [n = 217]	48.48 (5.40) [n = 32]	−0.57	38.78	0.57
	Severe	70.00 (11.69) [n = 28]	75.00 (8.84) [n = 2]	−0.75	1.23	0.57

*Note.* SD = standard deviation; HL = hearing loss; SO = significant other; Significance level for multiple comparisons with Sidak-Bonferoni corrections is *P*-level < 0.009.

### Statistical Analyses

The primary goal of the paper was to investigate whether an association exists between SO relationship type and hearing aid adoption. For model 1 the full sample (n = 47,167), discussed under “*Initial Data Preparation**”*** above, was used. Using SPSS, age (years) was entered into Block 1 of a hierarchical logistic regression analysis in SPSS, while sex (female or male) and SO relationship type (alone, carer, child, friend, parent, partner, or sibling) were entered into Block 2. The “alone” level operated as the reference level.

In order to replicate previous findings, two hierarchical binary logistic regression analyses were conducted by following the same protocol as performed in Singh and Launer (2016). Model 2 was conducted to investigate the association between hearing aid adoption and age, sex, and SO attendance status for the full sample (n = 47,167). Using SPSS, participant age (years) was entered in Block 1, while sex (female or male) and SO attendance status (alone or with a SO) were entered in Block 2. Model 3 was conducted on a subset of the sample that had available audiometric thresholds (n = 2,746) to investigate whether SO attendance status was associated with hearing aid adoption (yes or no) when also considering age, sex, audiometric hearing loss (WE4PTA) and the WE4PTA by SO attendance interaction. For model 3, age (years) and WE4PTA were entered in Block 1, while SO attendance status (alone or with a SO), sex (female or male), and the WE4PTA by SO attendance status interaction were entered in Block 2. It was hypothesized that findings from model 2 and model 3 would be consistent with Singh and Launer (2016), as described earlier.

For each of the logistic regression models, variables were retained in the model only if they had a p value of < 0.05. All predictor variables had a variance inflation factor of < 2.0, except for model 3, where the attendance of an SO (yes or no), and the interaction between the WE4PTA and the attendance of an SO, which were 17.59 and 17.90, respectively. By dropping the interaction term in model 3, it was possible to correct for multicollinearity, so this was done (i.e., model 4, which was identical to model 3 but without the interaction term). Unfortunately, by dropping the interaction term here, it was no longer possible to directly compare results to Singh & Launer (2016). The Pearson correlation between continuous predictors in model 3 was < 0.30. In order to assess goodness-of-fit, the Hosmer-Lemeshow test was used.

## Results

### Model 1: Age, Sex, and SO Relationship Type

Model 1 included the variables age, sex, and the type of SO relationship, and was statistically significant (−2 log likelihood = 58059.69, x^2^ (df = 8) = 1217.09, Nagelkerke R^2^ = 0.036, p < 0.0001). Goodness-of-fit was deemed inappropriate according to the Hosmer-Lemeshow statistic, which was significant (p < 0.0001). However, this is not of great concern, as it has been acknowledged by Hosmer and Lemeshow that for large sample sizes, this test may be significant even when the model fit is good (Hosmer, Hosmer, Le Cessie, & Lemeshow, 1997).

The Wald ratio for the coefficients associated with age (x^2^ (df = 1) = 823.70, p < 0.0001) and sex (x^2^ (df = 1) = 36.62, p < 0.0001) were both significant. Thus, the odds of adopting a hearing aid are significantly higher for participants who are older rather than younger (OR = 1.03; 95% CI [1.02, 1.03]), and they are significantly lower for patients who are male rather than female (OR = 0.88; 95% CI [0.85, 0.92]). The Wald ratio for the coefficient associated with the type of SO relationship was also significant, x^2^ (df = 6) = 213.48, p < 0.0001. Compared to attending alone, the odds of hearing aid adoption were significantly higher if a patient attended with their child (x^2^ (df = 1) = 48.86, p < 0.0001; OR = 1.42; 95% CI [1.29, 1.57]), parent (x^2^ (df = 1) = 11.59, p = 0.001; OR = 2.44; 95% CI [1.46, 4.09]), partner (x^2^ (df = 1) = 160.05, p < 0.0001; OR = 1.47; 95% CI [1.38, 1.56]), or sibling (x^2^ (df = 1) = 4.65, p = 0.031; OR = 1.41; 95% CI [1.03, 1.94]). It was also observed that the odds of adopting a hearing aid were not significantly different when attending with a carer (x^2^ (df = 1) = 1.56, p = 0.21; OR = 0.61; 95% CI [0.28, 1.33]) or a friend (x^2^ (df = 1) = 1.45, p = 0.23; OR = 1.13; 95% CI [0.93, 1.37]), compared to attending alone.

### Model 2: Age, Sex, and SO Attendance

Model 2 included the variables age, sex, and SO attendance as predictors, and was statistically significant (−2 log likelihood = 58074.87, x^2^ (df = 3) = 1201.91, Nagelkerke R^2^ = 0.035, p < 0.0001). Goodness-of-fit was deemed inappropriate according to the Hosmer-Lemeshow statistic, which was significant (p < 0.0001). As reported above for model 1, for large sample sizes such as this, the Hosmer-Lemeshow statistic may be significant even when the model fit is good (Hosmer, Hosmer, Le Cessie, & Lemeshow, 1997).

The Wald ratio for the coefficient associated with SO attendance at audiology appointments was statistically significant, x^2^ (df = 1) = 199.72, p < 0.0001. This means that the odds of hearing aid adoption for patients who attended with a SO were significantly higher than for those who attended alone (OR = 1.44; 95% CI = [1.37, 1.51]). The Wald ratios for age (x^2^ (df = 1) = 829.01, p < 0.0001), and sex (x^2^ (df = 1) = 35.01, p < 0.0001) were also significant. Therefore, the odds of hearing aid adoption for older participants was significantly higher than for younger participants (OR = 1.03; 95% CI = [1.02, 1.03]), and contrary to Singh and Launer (2016), the odds of hearing aid adoption were significantly lower for men than for women (OR = 0.89; 95% CI = [0.85, 0.92]; p < 0.001).

### Model 3: Age, WE4PTA, sex, SO Attendance, and WE4PTA by SO Attendance

Results for model 3 are influenced by multicollinearity, which suggests weakened statistical power and questionable statistical significance of predictor variables. However, results are reported here to compare against model 4, reported further below. Model 4 is the same as model 3, but without the interaction term, and results for remaining variables remained interpretively identical.

### Model 4: Age, WE4PTA, sex, and SO Attendance

Model 4 included the variables age, WE4PTA, sex, and SO attendance, as predictors, and was statistically significant (−2 log likelihood = 2555.03, x^2^ (df = 4) = 170.29, Nagelkerke R^2^ = 0.096, p < 0.0001). Goodness-of-fit was deemed appropriate according to the Hosmer-Lemeshow statistic, which was not significant, x^2^ (df = 4) = 11.74, p = 0.16.

The Wald ratio for the coefficients associated with age (x^2^ (df = 1) = 8.59, p = 0.003), WE4PTA (x^2^ (df = 1) = 77.13, p < 0.0001), and SO attendance (x^2^ (df = 1) = 32.56, p < 0.0001), were all significant. Thus, the odds of hearing aid adoption were significantly higher for individuals who were older compared to younger (OR = 1.01; 95% CI = [1.00, 1.02]), who had poorer compared to better audiometric hearing loss (OR = 1.04; 95% CI = [1.03, 1.05]), and for those who attended with a SO compared to those attended alone (OR = 2.47; 95% CI [1.82, 3.40]). The Wald ratio for the coefficient associated with sex (x^2^ (df = 1) = 2.85, p = 0.092; OR = 0.85; 95% CI [0.70, 1.03]) was not significant.

## Discussion

### Replication of Previous Research

Before discussing the primary goal, a secondary goal was to test whether results from Singh and Launer (2016) was reproducible, using new data. Just as was found in Singh and Launer (2016), we found that the odds of hearing aid adoption were greater when patients attended audiology appointments with a SO, and when patients were older rather than younger. Singh and Launer (2016) found that the effect of SO attendance on hearing aid adoption was more robust for individuals with less, compared to more, hearing loss, but unfortunately this effect could not be tested in the present study due to multicollinearity.

### Patient Hearing Aid Adoption Depends on Relationship Type of SO in Attendance

The primary goal of the current paper was to examine the association between the type of SO attending an audiology appointment and hearing aid adoption. After controlling for age and sex, a significant association was observed. This finding suggests that patient decision making about whether to adopt the use of hearing aids is significantly associated with the type of SO person who attends the appointment with them.

Specifically, compared to attending alone at their audiology appointments, the odds of patients adopting hearing aids were higher for those who attended with their child, partner, parent, or sibling, while the odds were not significantly different when attending with a carer or a friend. This result might be explained by the possibility that familial relationship ties tend to be stronger than non-familial relationship ties, and because family members may be more likely to be primary conversation partners. These results provide further evidence that social context and support from a SO positively impacts patients’ decision making for hearing aid adoption at audiology appointments. This new evidence is important because hearing aid owners take an average of 8.9 years to adopt after hearing aid candidacy is first determined (Simpson et al., 2019), and hearing aid non-owners report knowledge of having hearing loss for 10.5 years without yet adopting (Jorgensen & Novak, 2020). Given this new evidence of a positive association between attendance of familial SOs at audiology appointments and hearing aid adoption shown in this study, the previous recommendation from Singh & Launer (2016) to recommend SO attendance at hearing care appointments could now be extended to recommend that the SO be a familial connection, if possible.

On the other hand, these results also highlight the potential vulnerability of those patients who have been recommended a hearing aid (or HAs), but did not have familial support at their hearing care appointment. Some patients may not have familial connections, or may have relationships with SOs that are not supportive. Clinicians should try to be aware about the general nature of familial and non-familial relationship ties in their patients’ lives. For example, clinicians may need to implement more engaged follow-up discussions after a hearing aid recommendation, for those who have weaker, or only non-familial, relationship ties. Although more work needs to be done before recommendations can be made, who is in attendance at a patient's appointment can provide a simple cue to inform clinicians about what level of support they may need to provide for adequate hearing care.

### Limitations, Strengths, and Future Directions

The constraints associated with retrospective research designs are a notable limitation to the current study. For example, it was not possible to control which patients brought a SO to their appointment, as the data were analyzed well after collection. Hence, it is unclear whether SO attendance might influence hearing aid adoption for patients in a direct manner. Future work ought to implement an experimental design with random assignment for patients to either attend with an SO or alone to better understand the nature of this association. Additionally, due to the nature of these data, there is no available information regarding the perseverance of hearing aid adoption (e.g., hearing aid data logging, attendance of follow-up appointments, etc.). This point could not be stated more strongly: future work about hearing aid adoption should consider outcomes of hearing aid use after the decision to adopt hearing aids is made.

Another limitation was that clinicians did not follow the same approach when documenting the identities of the SOs in attendance because they were asked to do so using a free text description. Given the nature of free text data, it was necessary to develop a classification procedure that accounted for challenges like typos or spelling errors, capitalizations, and compounded responses like “brother John” instead of simply “brother”. For this reason, data cleaning was time consuming and resulted in data losses due to between-clinician differences in data reporting. Although the classification of SO type presented challenges, it may also be viewed as a strength of the current study as well as an example of the usefulness of data-driven retrospective analyses in general. It was possible to make use of available data to answer a relevant and novel research question. That said, future data-base related work on this topic should employ the use of pre-defined SO type classifications that clinicians could select from when defining the relationship of the SO to the patient during appointments. This would reduce ambiguity in response categories and increase data retention.

Clearly, the nature of SO relationships and their social support for patients is far more complex than this retrospective study could describe. For example, significant others who are adult children may not be the primary conversation partner of their parents who are patients. There are likely different dimensions related to SO attendance (e.g., strength of social support or the degree to which the relationship is embedded in a patients’ regular life, or the degree of relationship conflict surrounding communication problems) that are important to note, but were not measured in the current study. This highlights the need for future studies to a) directly measure relationship strength, and b) include other contextual factors that help to describe the relationship quality of patient-SO pair. A complementary variable of interest could be one that attempts to measure the degree of conflict arising from communication challenges due to a patient's hearing loss. For example, if there is conflict surrounding hearing challenges between a patient-SO pair, would the attendance of the SO help to unify the pair toward jointly addressing the communication issue by adoption hearing aids, or would this further exacerbate conflict between the pair? Furthermore, it may be helpful to develop a metric for the quality of the triadic patient-SO-clinician interaction to better understand and account for not only the presence of an SO at an appointment, but also perspectives about their presence from the triad (e.g., roles, contributions, etc.; Reynolds et al., 2019).

One other limitation is that the data for the present study were from private clinics only, thus patients who choose to adopt hearing aids would most likely be required to pay for them. It is often the case that people do not make big purchases without the presence of, or after discussion with, their SO, which may be a form of social influence not considered in our study. Also, it should be noted that the sample used in this retrospective study was from the United Kingdom, where citizens are eligible to receive free hearing aids from the NHS. These patients decided to attend appointments at a private clinic chain that offered free hearing tests. While hearing tests are also free through the NHS, patients may have opted to attend a private clinic for possible reasons such as faster accessibility to service, better geographic accessibility, and/or to obtain hearing instruments not provided via the NHS. Notably, investigations of patient data from private clinics should consider how factors, such as socio-economic status, may influence hearing aid adoption. Future work should consider collecting patient data from various clinic types, and/or for those who have financial coverage from a government or 3^rd^ party institution compared to those who pay privately.

A final note for future considerations relates SO involvement with the use of remote support in audiology such as synchronous and asynchronous tele-audiology or virtual care where clinicians may alter hearing aid settings at a distance. The rapid increase of virtual care appointments since the beginning of the covid-19 pandemic (Palmer, 2021) is a positive development for the field of audiology, but effort should be considered with regard to how technology developers and clinicians may involve SOs during virtual care appointments.

## Conclusion

This study used a large-scale data-driven approach to examine the association between the social context of real-world clinical audiology appointments and patient decision-making regarding hearing aid adoption. Although the mechanisms underlying this association remain unclear, it is promising that previous findings were replicated here using a large dataset, and showed a significant positive association between SO attendance at audiology appointments and hearing aid adoption. This study also suggests that the type of SO in attendance at audiology appointments may influence the likelihood of hearing aid adoption. Specifically, the attendance of familial SO relationship types (i.e., child, parent, partner, or sibling) was associated with greater odds for patients to adopt hearing aids while the attendance of non-familial SO relationship types (i.e., friends, carers) was not. Although more work needs to be done to better understand the nature of patient-SO relationships (e.g., is the SO also the primary conversation partner?) and their association with hearing aid adoption (i.e., is the relationship causal?), the previous recommendation from Singh & Launer (2016) which suggested that clinicians should consider the appropriateness of encouraging SOs to attend audiological appointments from an early stage in the hearing care pathway. Furthermore, clinicians should not hesitate to ask about the strength of relationship ties in patients’ lives, and to follow-up more diligently about hearing aid recommendations for those who have less relationship support.
